# Withdrawal from escalated cocaine self-administration impairs reversal learning by disrupting the effects of negative feedback on reward exploitation: a behavioral and computational analysis

**DOI:** 10.1038/s41386-019-0381-0

**Published:** 2019-04-06

**Authors:** Peter Zhukovsky, Mickael Puaud, Bianca Jupp, Júlia Sala-Bayo, Johan Alsiö, Jing Xia, Lydia Searle, Zoe Morris, Aryan Sabir, Chiara Giuliano, Barry J. Everitt, David Belin, Trevor W. Robbins, Jeffrey W. Dalley

**Affiliations:** 1 Department of Psychology, University of Cambridge, Downing Street, Cambridge, CB2 3EB UK; 2 Behavioural and Clinical Neuroscience Institute, University of Cambridge, Cambridge, CB2 3EB UK; 3 Department of Psychiatry, University of Cambridge, Cambridge, CB2 2QQ UK

**Keywords:** Risk factors, Decision

## Abstract

Addiction is regarded as a disorder of inflexible choice with behavior dominated by immediate positive rewards over longer-term negative outcomes. However, the psychological mechanisms underlying the effects of self-administered drugs on behavioral flexibility are not well understood. To investigate whether drug exposure causes asymmetric effects on positive and negative outcomes we used a reversal learning procedure to assess how reward contingencies are utilized to guide behavior in rats previously exposed to intravenous cocaine self-administration (SA). Twenty-four rats were screened for anxiety in an open field prior to acquisition of cocaine SA over six daily sessions with subsequent long-access cocaine SA for 7 days. Control rats (*n* = 24) were trained to lever-press for food under a yoked schedule of reinforcement. Higher rates of cocaine SA were predicted by increased anxiety and preceded impaired reversal learning, expressed by a decrease in lose-shift as opposed to win-stay probability. A model-free reinforcement learning algorithm revealed that rats with high, but not low cocaine escalation failed to exploit previous reward learning and were more likely to repeat the same response as the previous trial. Eight-day withdrawal from high cocaine escalation was associated, respectively, with increased and decreased dopamine receptor D2 (*DRD2*) and serotonin receptor 2C (*HTR2C*) expression in the ventral striatum compared with controls. Dopamine receptor D1 (*DRD1*) expression was also significantly reduced in the orbitofrontal cortex of high cocaine-escalating rats. These findings indicate that withdrawal from escalated cocaine SA disrupts how negative feedback is used to guide goal-directed behavior for natural reinforcers and that trait anxiety may be a latent variable underlying this interaction.

## Introduction

Despite considerable research, the psychological mechanisms underlying the maladaptive behavior of individuals addicted to drugs remain poorly understood, in particular the propensity of such individuals to continue taking drugs despite mounting negative impacts. A disregard for harmful consequences implies an innate or acquired imbalance in how positive and rewarding outcomes are perceived and processed relative to punishment signals. Consistent with this view, rats exposed to cocaine fail to utilize outcome value to guide behavior [1] and continue to seek drugs despite their devaluation [2–4] or the risk of punishment [5–7]. The neural mechanisms underlying this maladaptive behavior are not well understood but may underlie compulsive forms of drug seeking [8].

Neural activity in the orbitofrontal cortex (OFC) is broadly acknowledged to represent outcome value and expectation used to guide value-based decision-making [9–15]. The OFC also plays a key role in behavioral flexibility, the capacity to rapidly track changing stimulus-response contingencies in reversal learning procedures [16–19], and structural and functional changes in the OFC are present in individuals addicted to drugs [20–26]. Consistent with these findings, reversal learning is impaired in rats and monkeys exposed to cocaine [17, 27–29]. Thus, drug-induced abnormalities in OFC networks that include the amygdala and striatum [11] may underlie the inflexibility and insensitivity to outcomes associated with drug exposure [1].

The ability to respond flexibly to changing stimulus-response contingencies requires an animal to learn about the prospective values of the responses using both positive and negative feedback. Reversal learning tasks require animals to optimize their choice strategy to maximize the rewards they obtain, while at the same time occasionally exploring alternate reward options. Using the representation of predictive relationships in the environment, acquired through trial and error, reversal learning requires animals to switch responding to a now correct stimulus while ignoring the interference of a recently rewarded, but now no-longer-correct stimulus. Reinforcement learning has been proposed as a tractable computational process underlying trial-and-error learning [30, 31] with utility in modelling aspects of addiction [32, 33] and stimulant administration in rodents [34, 35]. Indeed, computational psychiatry has become an increasingly popular translational methodology to investigate mental health [36, 37], especially if the computational models are constrained by neurobiological data [38].

In this study we therefore used behavioral and computational methods to define the nature of reversal learning deficits in rats with a history of escalated cocaine self-administration, compared with food-reinforced, cocaine-naïve rats. We hypothesized that cocaine-exposed rats would be less sensitive to negative feedback in a spatial-discrimination reversal learning task than drug-free controls. We further evaluated possible modulatory effects of trait anxiety given that this predicts the individual propensity to escalate cocaine SA under long-access conditions [39–41], as well as response perseveration in a spatial reversal learning task [42]. Finally, we measured ex vivo gene transcript levels of dopamine (DA) and serotonin (5-HT) receptors in the OFC and striatum as neural correlates of cocaine-induced impairments in reversal learning.

## Materials and methods

### Subjects

**Fig. 1:**
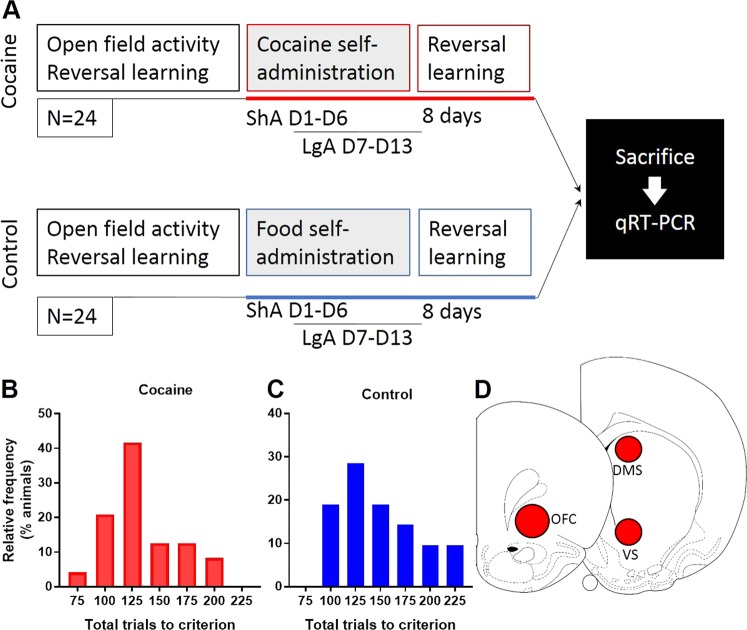
**a** Experimental timeline. Two cohorts of rats (each *n* = 24) were assessed for open field activity as a measure of anxiety followed by spatial-discrimination reversal learning. Rats in cohort 1 were trained to intravenously self-administer cocaine under short- and long-access schedules (ShA; LgA) while rats in cohort 2 (control group) responded for food reinforcement under identical schedules. Finally, rats in both cohorts were re-assessed for reversal learning prior to sacrifice and post mortem qRT-PCR. **b**, **c** Frequency distribution plots of ‘total trials to criterion’ for the cocaine and control rats. **d** qRT-PCR was used to assess gene expression in the OFC, dorsal and ventral striatum

Subjects were male Lister-hooded rats (*n* = 48) weighing 280–300 g at the beginning of the experiments (Charles River, Kent, UK). Rats were maintained at 85–95% of free-feeding weights. Each animal received 18 g of food chow once a day within 2 h after behavioral testing and had ad libitum access to water. When no behavioral training or testing took place, rats received 20 g of chow per day. Rats were either housed in groups of four or singly after catheter implantation and during the cocaine self-administration experiment under a reversed 12 h light/dark cycle (lights off 07:00 h until 19:00 h). Two cohorts of rats were trained and tested on a spatial-discrimination serial reversal learning task [42, 43] prior to the assessment of anxiety on an open field test (Fig. 1a). Rats in cohort 1 (*n* = 24) were trained to self-administer intravenous (i.v) cocaine (six daily short-access sessions; seven daily long-access sessions) whereas cohort 2 rats (*n* = 24) were trained to lever-press for food pellets (Noyes dustless pellets, 45 mg, Sandown Scientific, UK) over an equivalent period of days. Rats from both cohorts were re-tested on the reversal learning task 8 days after cocaine or food self-administration. All experiments complied with the statutory requirements of the Animals (Scientific Procedures) Act 1986 following local ethical review by the University of Cambridge Animal Welfare and Ethical Review Body (PPL 70/8072).

### Behavioral assessment

#### Reversal learning

Spatial-discrimination reversal learning was assessed using 12 5-choice operant chambers placed in ventilated, sound-attenuating cubicles (Med Associates, Georgia, VT), as previously described [42, 43]. Subjects were initially habituated to the apparatus over 2 days, with each session lasting 20 min. They were then trained to enter the magazine to trigger the illumination of a single stimulus light (left or right) and to respond in one of the two illuminated apertures for food delivery under a fixed-ratio (FR) 1 schedule of reinforcement. Once rats had achieved 50 correct responses, food reward was successively delivered under FR2 and FR3 schedules to the same criterion within a 30 sec limited hold period. Failure to respond within the 30 sec period resulted in a 5 sec time-out. Once rats were able to achieve criterion under a 5 sec inter-trial interval, they were tested for spatial discrimination followed the next day by a reversal of the stimulus-reward contingency. Firstly, rats were given a maximum of 1 h to complete the discrimination task by achieving nine correct trials across the previous 10 trials. Once rats achieved this criterion and consistently responded at the rewarded (left or right) aperture, the session ended. On the following day, rats were given a retention test of the discrimination learned on the previous day, in which the same aperture was rewarded. Once rats achieved criterion (9/10 correct), they then completed three reversals (Fig. 3g). This test session lasted for ~1 h. Following each contingency reversal, responses in the previously incorrect aperture were signaled as correct (and reinforced with a food pellet) whereas responses in the previously correct aperture were signaled as incorrect (and not reinforced with food). Two rats (one in the cocaine and one in the control group) who failed to achieve the criterion of three successful reversals were excluded from the analysis. In addition, five rats in the cocaine group were excluded from the study due to suspected catheter failure. Following cocaine or food self-administration, rats were re-trained over five sessions to respond for food on the spatial-discrimination task under a FR3 schedule of reinforcement. On the test day, rats were given a retention test prior to completing three reversals, identical to the procedure described above.

#### Anxiety assessment

A black, matte arena of 150 cm diameter and 50 cm high walls was used to assess behavior in the open field [44] under white lights (70 lux). The central area of the arena was defined as a circle with a diameter of 75 cm. Exploratory behavior in the maze was recorded and monitored on a ceiling-mounted Yi Action Camera (Xiaomi, Japan) connected to a computer via Wi-Fi and analyzed using Icarus V2.09 (University of Manchester, UK 2002–2003) software. Rats were placed in the center of the arena with behavior recorded for 8 min. The arena was cleaned with water between each animal. An anxiety score was calculated as the proportion of time spent in the center of the arena in the total time of 8 min.

#### Intravenous cocaine self-administration

Twenty-four operant chambers (31.8 cm long × 25.4 cm width × 34.3 cm high), constructed of Plexiglas and a metal grid floor, were each placed in ventilated, sound-attenuating cubicles (Med Associates, Georgia VT). Whisker Control software (Second Order, Cardinal and Aitken, 2010) controlled the apparatus. Two retractable levers and a white light emitting diode located above each lever were placed along one wall of the chamber, with a house-light positioned on top of the opposite wall. Cocaine infusions were delivered via implanted intravenous-dwelling catheters connected to a syringe-driven infusion pump (Semat Technical, Herts, UK) and Tygon tubing. Infusions were delivered at a rate of 20 μl/sec. Each infusion contained 0.25 mg cocaine hydrochloride.

A single-lumen catheter (CamCath^®^, Cambridge, UK, inner diameter 0.28 mm; outer diameter 0.61 mm; dead volume 12 μl) was implanted in the right jugular vein under ketamine hydrochloride (100 mg/kg, intraperitoneal, Ketaset) and xylazine (9 mg/kg, i.p., Rompun) anesthesia. The proximal end of the silastic catheter was inserted in the right atrium and the distal end was sutured subcutaneously between the scapulae. To prevent infection, rats were treated with a subcutaneous injection of 10 mg/kg Baytril^®^ (Genus Express, Bury St. Edmunds, UK) on the day before surgery and were then given 10 mg/kg Baytril in mashed pellets for 5 days post-surgery. Following surgery, catheters were flushed daily with saline-heparin (100 IU/ml), with a recovery period of 10 days.

Rats acquired cocaine SA during six daily 1 h long sessions (short-access; ShA), under a fixed-ratio (FR)-1 schedule of reinforcement, and each 0.05 ml infusion containing 0.25 mg cocaine hydrochloride (MacFarlan, UK) was delivered over 5.7 s. Thereafter, rats were given long-access (LgA) exposure to cocaine over seven daily 6 h long sessions. Catheters were flushed with saline-heparin before and after each session. Each session started with the chamber being illuminated and the 2 levers inserted. Active lever presses resulted in a cocaine infusion and a white cue light cue for 5 sec followed by a 20 sec time-out period, during which both levers were retracted. Inactive lever presses had no scheduled consequences. Active and inactive levers were randomly assigned to the 24 rats.

#### Food reinforcement

Twelve operant chambers of the same configuration and manufacturer as the cocaine SA chambers were used. These only differed by the presence of food pellet dispenser and magazine. Rats (*n* = 24) were trained to make a lever-press response for a single food pellet (Noyes dustless pellets, 45 mg, Sandown Scientific, UK) under an FR-1 schedule for the first 6 daily 1 h sessions. Thereafter, rats responded under an FR5 schedule for the remaining 7 days. Rats in this group did not have a surgically-implanted i.v. catheter. In order to maximize the time spent in the testing context, and in accordance with the cocaine SA experiment, the post-reinforcement time-out period was set to 60 sec. Inactive lever responses were recorded but had no programmed consequences. The maximum number of pellets available was adjusted to match the number of lever-press responses made by the cocaine SA rats. Since rats consumed the food pellets whenever they became available, the maximum number of pellets determined the session duration.

### Computational modeling

Several learning models were used to simulate the reversal learning data, including three variants of the Q-learning model [45], defined below and the three parameters: *α*, *β*, and *κ*. Model parameters were fitted to each animal’s reversal data individually and then compared using analysis of variance (ANOVA). The learning rate *α* determines how quickly the model adjusts to the expected value of a response following positive or negative feedback. High α values allow the agent to increase (or decrease) the expected Q-value placed on that response if the response is followed by a reward (or not). The inverse temperature parameter *β* regulates how much an agent explores by responding randomly or exploits what the agent learned about the responses to date. A low *β* value would lead an agent to rely on the expected Q-values of the responses and hence exploit what they have learnt about the responses already. A high *β* value would lead to exploration that under some circumstances may lead to higher rewarded outcomes. However, in the present reversal task, with deterministic outcomes, a high *β* value would result in fewer rewards. Finally, the choice autocorrelation parameter *κ* is a measure of “stickiness”, or how likely an animal will perform the same response again regardless of reward outcome. Values of *κ* close to 1 reflects an agent “sticking” to the previous response while *κ* values close to −1 reflects choice alternation.

#### Model-free Q-learning: model 1

Simple Q-learning is equivalent to Rescorla-Wagner learning [30] whereby an agent assigns an expected Q-value to each choice available; presently a left or right response (L or R) at each trial *t*. The expected Q-value is updated on each trial according to the following:

$$P\left( c_t = L| Q_t\left( L \right), Q_t \left( R \right)\right) = \frac{\exp \left( {Q_t \left( L \right)/ \beta} \right)}{\exp \left( {{Q_t \left( L \right)/ \beta}} \right) + \exp \left( {Q_t \left( R \right)/ \beta} \right)}$$

where 0 ≤ *α* ≤ 1 is a learning parameter, *Q*_*t*_(*c*_*t*_) is the value of the choice *c*_*t*_ at trial *t* and *r* takes the value of 1 if the choice was rewarded and a value of 0 if not. A large *α* implies faster updating of the expected Q-values of a response after a trial is completed. The probability of making the choi*c*e *c*_*t*_ at trial *t* was calculated using the *softmax* rule:

$$P\left( {c_t{\mathrm{ = }}L\left| {Q_t\left( L \right),Q_t\left( R \right)} \right.} \right) = \frac{{\exp \left( {\beta \ast Q_t\left( L \right)} \right)}}{{\left. {\exp \left( {\beta \ast Q_t\left( L \right)} \right){\mathrm{ + }}\exp \left( {\beta \ast Q_t\left( R \right)} \right.} \right)}}$$

where *β* is the inverse temperature parameter, with larger *β* values leading to more exploration of the responses with lower Q-values. On the other hand, smaller *β* values result in exploitation of the response with higher Q-values.

#### Model-free Q-learning: model 2

Model 1 was extended to include a separate *α* for learning from rewards and losses, *α*_*REWARD*_ and *α*_*NO REWARD*_, depending on whether the animal received a reward on trial *t*. The decision probability was updated in the same way as in Model 1.

#### Model-free Q-learning: model 3

A different variation of Model 1 included only one learning parameter *α* as in Model 1, but an additional autocorrelation parameter in the observational part of the model:

$$	P\left({ c_t = L\left| {Q_t\left( L \right),\,Q_t\left( R \right),\,L_{t - 1},\,R_{t - 1}} \right.} \right) \\ 	\quad\quad=\frac{{{\mathrm{exp}}\left( {Q_t\left( L \right)}/{\beta + \kappa \ast L_{t - 1}} \right)}}{{{\mathrm{exp}} \left({Q_t\left( L \right)}/ {\beta + \kappa \ast L_{t - 1}} \right) + {\mathrm{exp}}\left({Q_t\left( R \right)}/ {\beta + \kappa \ast R_{t - 1}} \right)}}$$

whereby a larger *κ* results in greater probability of the choice *c*_*t*_ at trial *t* being the same as the choice *c*_*t*_ at trial *t−1*. The same approach was applied to the right sided choice.

#### Model fitting

The probability of *Data D* (a sequence of choices and rewards) is the product of the individual probabilities of making a choice *c*_*t*_ at trial *t*:

$$\begin{array}{l} P\left( {{\mathrm{Data}}\,D\left| {{\mathrm{Model}}\,M,\,{\mathrm{parameters}}\,\theta } \right.} \right) = P\left( {D\left| {M,\,\theta } \right.} \right){\mathrm{ = }}{\prod} {P\left( {c_t{\mathrm{|}}Q_t\left( L \right),\,Q_t\left( R \right)} \right)} \end{array}$$

Model space was treated as discrete, using the following range of parameters: 0.001 ≤ *α* ≤ 1 with a step size of 0.08; 0.005 ≤ *β* ≤ 5 with a step size of 0.08 and −1 ≤ *κ* ≤ 1 with a step size of 0.08. Parameter range was chosen based on the *a* priori expectations regarding *α* and *κ*, as well as empirical information about best fit *β* parameters. Best fit parameters ($\widehat \theta _M$) were chosen to maximize the log-likelihood of the observed data for each participant over all parameter sets (*θ*) by finding the maximum of the probability density function, $\arg \mathop {\mathrm{max }}\limits_\theta P\left( {D\left| {M,\,\theta } \right.} \right)$.

#### Model comparison

Nested models were compared using the likelihood ratio test that contrasts the log-likelihood of the data given the best fit parameters ($\widehat \theta _M$):

$$d{\mathrm{ = }}2 \ast \left[ {\log P\left( {D\left| {M_2,\,\widehat \theta _{M_2}} \right.} \right) - {\mathrm{log}}\log P\left( {D\left| {M_1} \right.,\widehat \theta _{M_1}} \right)} \right]$$

As *d* follows the χ-square distribution, the difference in data likelihood associated with increasing the number of parameters from two (*α*, *β*) to three (*α*_*REWARD*_, *α*_*NO REWARD*_, *β* or *α*, *β*, *κ*) is significant at *p* = 0.05 for *d* > *3.842*. An example of model predicted probability of choosing left or right (for $\widehat \theta _M$) together with the sequence of observed responses and rewards is shown in Fig. 3g. A biased measure of model fit, *pseudo r*^*2*^, was computed as follows:

$${\mathrm{pseudo}}\,r^2{\mathrm{ = }}\frac{{\log P\left( {D\left| {M,\,\widehat \theta _M} \right.} \right) - 0.5^n}}{{0.5^n}}$$

where *n* represents the number of trials and the probability of observing the data when the best fit parameters are contrasted against the probability of observing the data at random (0.5^*n*^). Although *pseudo r*^*2*^ will increase with the number of parameters fitted and does not penalize overfitting, it can be useful in linking the modelling results to more traditional statistical methods such as linear regression. Finally, the Bayesian Information Criterion (BIC) provided an alternative measure of model fit:

$${\mathrm{BIC}}{\mathrm{ = }}{\mathrm{log}}\left( {P\left( {D\left| M \right.} \right)} \right) \approx {\mathrm{log}}\left( {P\left( {D\left| {M,\widehat \theta _M} \right.} \right)} \right) - \frac{n}{2}{\mathrm{log}}\,m$$

where *n* = number of free parameters and *m* = number of observations. We implemented this analysis using in-house Matlab scripts (R2016a), which can be found in the following link: https://github.com/peterzhukovsky/reversal_learning).

### Postmortem gene expression

Aliquots of brain tissue (diameter 1.0 mm) were extracted from 150 μm frozen slices. Their location is shown in Fig. 1d. miRNeasy Mini kit (Qiagen, UK) with additional DNAse digestion was used to extract RNA from the frozen samples. RNA yields were quantified using a Nanodrop 2000 spectrophotometer (Thermo Fisher, UK). First-strand cDNA was synthesized from 5 ng total RNA using random hexamer primers from the RevertAid First-Strand cDNA Synthesis Kit (Thermo Scientific, UK) and diluted to 2.5 ng per μl. SYBR green-based quantitative real-time polymerase chain reaction (qRT-PCR) was performed on the CFX96 Touch Thermal Cycler (Bio-Rad, UK). PCR on duplicates was performed using 0.25 mM of each primer. Efficiencies were calculated using linregPCR and the ΔΔCt method [46], normalizing against two reference genes (Tubulin and Β-Actin) and the mean of the food control group. Primer pairs were purchased from Sigma–Aldrich, as detailed previously [43]. PCR runs were set up as follows: 95 °C for 5 min; 40 cycles at 95 °C for 10 s; 60 °C for 10 s, and 72 °C for 1 min.

### Statistical analyses

All statistical analyses were carried out using SPSS (IBM version 23). Rats assigned to the cocaine SA experiment were segregated into two groups (*n*_*1*_ = 9; *n*_*2*_ = 10) using a median split based on the escalation ratio, defined as the proportion of infusions taken on the last 2 days of LgA to the infusions taken on the first day of LgA. A mixed-effects ANOVA with session (13 levels) and cocaine escalation group (High vs Low) as within- and between-subject factors, respectively, was used to confirm the different cocaine self-administration profiles. Further, two-way ANOVAs were used to assess the effect of group (controls vs high vs low cocaine escalation) on reversal performance, including the total number of trials to reach criterion, the number of perseverative errors (7/10 incorrect) to criterion; lose-shift and win-stay probabilities; alpha, beta and kappa model parameters. While group was used as a between-subject factor, time of testing (at baseline or post-cocaine/food SA) was used as within-subject factor. A mixed-effects two-way ANOVA was used to test for the between-subject effects of group (controls, HE, and LE) and the within-subject effect of region (OFC, VS, and DMS) on mRNA expression of each mRNA receptor subtype (DRD2, DRD1, HT2AR, and HT2CR). LSD tests were chosen for post hoc comparisons due to the increased power. If sphericity was violated as indicated by Mauchly’s test, a Greenhouse-Geisser correction was used. Linear regressions were used to test for associations between reversal learning, cocaine escalation, and anxiety scores. Statistical significance threshold was set at *p* < 0.05.

## Results

### High escalation of cocaine SA impairs reversal learning following 8 days of withdrawal

**Fig. 2:**
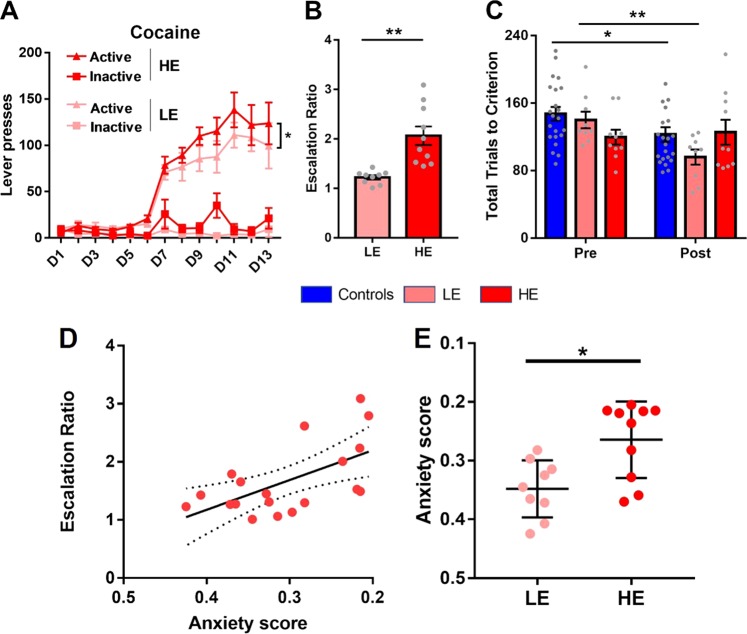
**a** Active and inactive lever-press responses of rats trained to self-administer cocaine. Data shown are means ± SEM. Since rats responded on a fixed-ratio 1 schedule, the number of lever presses was equivalent to the number of infusions received. Rats were divided into two groups: high escalation (HE) and low escalation (LE), based on a median split of escalation ratios. The escalation ratio was calculated as the ratio of the average number of active lever responses on days 12 and 13 to the number of lever responses on day 7 (D7—the first long-access session). During the first 6 days rats were given short-access to cocaine (1 h daily sessions) under a fixed-ratio-1 (FR-1) schedule of reinforcement. On days 7–13 inclusive, access to cocaine was increased to 6 h under an FR-1 schedule. **b** Escalation ratios for each animal in the high and low escalation groups, based on a median split (independent samples t_17_ = 4.2, *p* = 0.0006). **c** Individual reversal learning scores (total trials to criterion) before and after cocaine exposure in LE and HE rats compared with control rats. Data are means ± SEM. *p < 0.05. **p < 0.01. Relationships between anxiety and escalation of intravenous cocaine self-administration are shown in plots (**d**, **e**), including a line of best of fit with 95% confidence intervals in dotted lines. A lower anxiety score equates to increased anxiety in the open field arena. **d** Significant positive relationship between escalation ratio during the first hour of cocaine self-administration and anxiety scores (*r*^*2*^ = 0.29, p < 0.05), consistent with significant group differences in anxiety scores between LE and HE rats (**e**)

Following the assessment of reversal learning and anxiety, rats acquired i.v. cocaine SA over 6 consecutive days (D1-D6), as shown in Fig. 2a. Over the 13 days of cocaine SA rats responded differentially on the active and inactive levers and in response to increased cocaine availability (D7-D13) increased their responding for cocaine as shown by a significant increase in the number of active lever-press responses during this period (F_2.93,132_ = 5.0, *p* = 0.004, η^2^ = 0.19). Two groups of rats were subsequently formed—low escalation (LE) and high escalation (HE)—based on a median split of escalation ratio, calculated as the mean number of infusions during days 12 and 13 divided by the number of infusions on day 7 (i.e., the first day of long-access exposure). The mean (±1SEM) escalation ratio for LE and HE rats was 1.2 ± 0.04 and 2.1 ± 0.19, respectively (Fig. 2b). However, the groups did not differ in terms of the total amount of cocaine taken during the LgA sessions (total of 165.4 mg/kg/rat and 176.4 mg/kg/rat for LE and HE rats, respectively, *p* > 0.6). A separate group of control rats (*n* = 23) responded for food reinforcement and were matched to the cocaine group in terms of the maximum number of lever-press responses they could make.

We next assessed whether variation in reversal learning predicted cocaine escalation and, in turn, what effect long-access cocaine exposure had on reversal learning itself, measured 8 days after the end of self-administration. As shown in Fig. 2c, rats were generally faster to reverse when assessed for the second time on the reversal learning task (i.e., made fewer trials to criterion). Thus, a mixed-effects ANOVA with exposure time (pre- versus post-food/cocaine) and group (control, LE, and HE) as within- and between subjects factors, respectively, revealed a significant main effect of exposure time (F_1,38_ = 7.74, *p* = 0.008) and a trend for an interaction between group and exposure time (F_2,38_ = 3.1, *p* = 0.056). Post-hoc LSD tests revealed that while the number of trials to criterion significantly decreased during the second (‘post’) assessment in control and LE rats, this was not the case in HE rats. No significant differences between the three groups were found at baseline or post-cocaine (post hoc LSD, *p* > 0.05).

An analysis of perseverative errors revealed an interaction between time and group (F_2,38_ = 3.3, *p* = 0.047). The low escalation group improved over time (LSD, *p* = 0.007), resulting in significant group differences between LE and HE (LSD, *p* = 0.035) and LE and controls (LSD, *p* = 0.049) during the second assessment. However, baseline (i.e., ‘pre’) levels of perseverative responding were not significantly different between control, LE, and HE rats. These findings indicate that rats with a history of escalated cocaine intake (HE) failed to show the expected improvement in behavioral flexibility after repeated testing on the reversal learning task.

### Anxiety but not reversal learning predicts cocaine escalation

Figure 2 summarizes the dimensional relationships of anxiety with cocaine escalation. Anxiety was positively related to the escalation of cocaine SA (F_1,17_ = 9.3, r^2^ = 0.354, *p* = 0.007, Fig. 2d). Thus, anxiety scores were significantly different between future LE and HE rats (Fig. 2e). However, using linear regression models, we found neither a relationship between baseline behavioral flexibility (total trials to criterion) and escalation ratio (r^2^ = 0.01, *p* > 0.05, supplementary fig. 1A) nor a significant relationship between anxiety and behavioral flexibility (r^2^ < 0.06, *p* > 0.05, supplementary fig. 1B).

### High cocaine escalation decreases exploitation of previously learnt choice values and increases choice autocorrelation

Adding a third parameter in models 2 and 3 significantly improved the model fit compared with model 1. Model 3 provided a better fit of the data derived from the cocaine group than model 2 (*average pseudo r*^*2*^ = 0.16 compared to *pseudo r*^*2*^ = 0.14, and average BIC = 66.2 compared to average BIC = 67.6, respectively) while model 2 provided a better fit of the data derived from the control group than model 3 (*pseudo r*^*2*^ = 0.20 compared to *pseudo r*^*2*^ = 0.21, and average BIC = 69.8 compared to average BIC = 70.3, respectively). Model 3 was therefore chosen as the preferred model given its superiority in modeling the post-cocaine data, the main dataset of interest, and as a means to assess choice autocorrelation. A fourth model was also tested that included four parameters: a reward learning rate, a non-reward learning rate, beta, and kappa. This model failed to improve upon the fit of model 3 and hence was not included in the analysis (Supplementary Table 1).

**Fig. 3:**
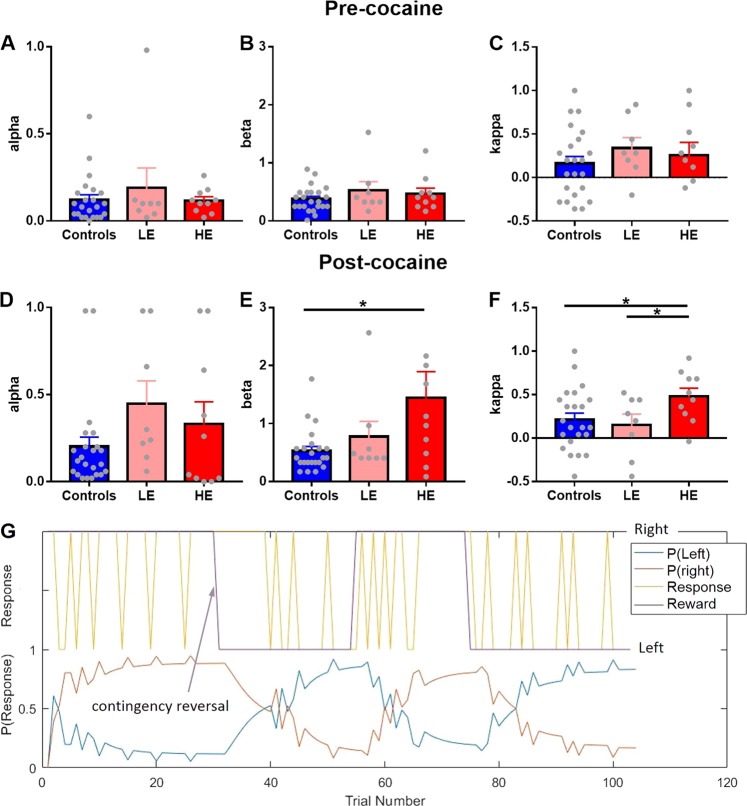
Modelling variables of learning and response flexibility on the spatial reversal learning task before and after intravenous cocaine self-administration compared with control rats. Data are means ± 1SEM. No significant differences in α, β and κ were observed in future LE and HE rats compared with control rats (**a**–**c**, respectively). Whereas the rate of learning of a response after the completion of each trial (α) was not significantly affected by cocaine exposure (**d**), a significant increase in β (**e**) and κ (**f**) was observed in HE rats (*p < 0.05; **p < 0.01). Thus, HE rats failed to exploit what they had previously learnt (increased β) and showed an increased tendency to make the same response as the previous trial (increased κ). An example of the model fit is shown in the lower panel (**g**) with individual left and right responses in the upper yellow traces alongside the rewarded side (violet trace) and in the lower trace the modelled probabilities of the same animal making a left or right response using the modelled values of α, β and κ

Figure 3 reports individual modeled parameters for control, LE, and HE rats before and after cocaine SA. In addition to a significant main effect of time (F_1,38_ = 9.5, *p* = 0.004) and group (F_1,38_ = 4.7, *p* = 0.015), a significant interactive effect of group (controls vs LE vs HE) and time (pre vs post-cocaine) was found on beta (F_2,38_ = 3.3, *p* = 0.048) but not on alpha and kappa (F_2,38_ = 1.2, *p* = 0.33, F_2, 38_ = 2.2, *p* = 0.13). Post hoc comparisons revealed no significant group differences in *α*, *β*, or *κ* prior to cocaine exposure (all *p* > 0.3, Fig. 3a–c). However, following cocaine self-administration, HE rats showed a significantly increased *β* value (lower exploitation, Fig. 3e) compared with controls (LSD, *p* *=* 0.0002) and LE rats (*p* = 0.024) together with a significantly increased *κ* value (an increased tendency to repeat the last response, Fig. 3f) compared with control and LE rats (*p* = 0.049). Importantly, neither LE nor HE rats differed from controls in the rate of learning parameter, *α*, after cocaine SA (*p* > 0.1, Fig. 3d).

### Cocaine exposure has differential effects on lose-shift and win-stay behavior

**Fig. 4:**
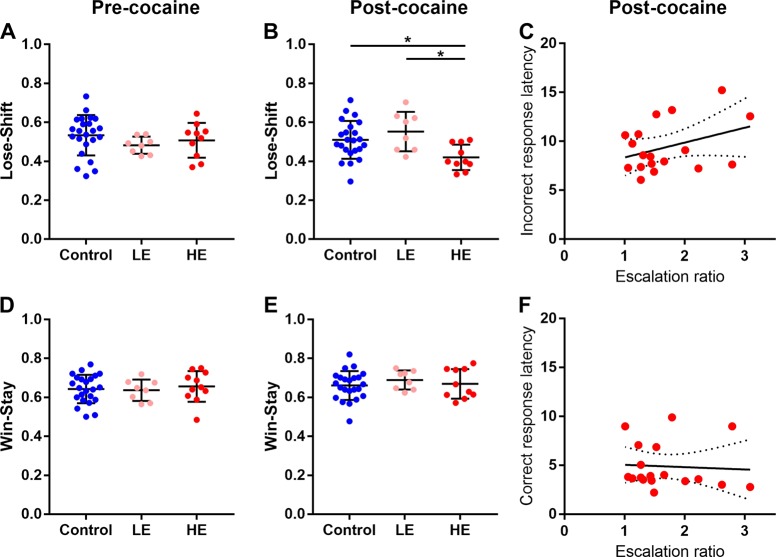
Lose-shift and win-stay probabilities on the spatial reversal learning task before and after intravenous cocaine self-administration compared with control rats. Data are means ± SEM. Prior to cocaine exposure there were no significant differences in lose-shift and win-stay probabilities between any of the groups (**a**, **d**). However, in rats exhibiting high escalation, lose-shift probabilities significantly decreased compared with low escalation and control rats (**b**) unlike win-stay probabilities (**e**). Escalation ratios did not significantly correlate with incorrect (**c**) or correct (**f**) response latencies. Shown are the lines of best fit (solid lines) and 95% confidence intervals (dotted lines)

A mixed effect ANOVA revealed a significant interactive effect of time (‘pre’ vs ‘post’) and group (controls vs LE vs HE) on lose-shift probability (F_2,38_ = 5.2, *p* = 0.01, Fig. 4), but not on win-stay probability. Post hoc LSD tests revealed that this effect was driven by a significant decrease in lose-shift probability in HE rats (LSD, *p* = 0.014) compared with control and LE groups (LSD, *p* = 0.004, Fig. 4b) and notably was not present before the rats were exposed to cocaine (Fig. 4a). In contrast, win-stay probability was unaffected by cocaine exposure (Fig. 4e) and was no different between control, LE, and HE rats prior to cocaine SA (Fig. 4d). Using linear models, we found no significant relationship between escalation ratio, assessed over 6 h sessions, and incorrect response latencies, defined as time to initiate a new trial after the end of the previous trial, (r^2^ = 0.13, *p* = 0.14, Fig. 4c) or correct response latencies (r^2^ = 0.06, *p* = 0.8, Fig. 4f).

### Differential effects of cocaine on the expression of genes encoding DA and 5-HT receptors

**Fig. 5:**
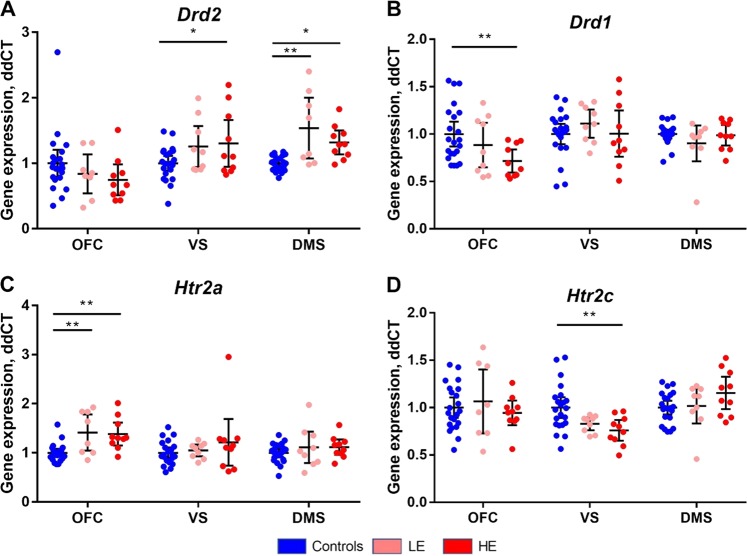
mRNA expression of *DRD2* (**a**) *DRD1* (**b**), *HTR2A* (**c**), and *HTR2C* (**d**) in the orbitofrontal cortex (OFC), ventral striatum (VS), and dorsomedial striatum (DMS) of control (*n* = 23), LE (*n* = 9), and HE (n = 10) rats. *p < 0.05 versus controls. Data are means ± 95% CIs

Figure 5 shows gene transcription levels of candidate DA and 5-HT receptors in the OFC, ventral striatum (VS), and dorsomedial striatum (DMS). Two-way ANOVA with group (control, LE, and HE) and region (OFC, VS, and DMS) revealed significant interactions between region and group for *DRD2* (F_4,113_ = 4.6, *p* = 0.002, Fig. 5a) and *HT2CR* (F_4,113_ = 3.2, *p* = 0.017, Fig. 5d), but not

*DRD1* (F_4,82_ = 2.3, *p* = 0.06, Fig. 5b) and *HT2AR* (F_4,82_ = 1.5, *p* = 0.20, Fig. 5c). Post hoc LSD contrasts revealed significant increases in *DRD2* expression in the VS of the HE group and in the DMS of LE and HE groups compared with the control group. *DRD1* expression in the OFC significantly decreased in the HE groups compared with the control group, whereas *HTR2A* expression increased significantly in both escalation groups in the OFC compared with controls. *HTR2C* expression was significantly decreased in the VS of HE rats compared with controls.

## Discussion

Our findings demonstrate several features and consequences of long-access cocaine self-administration that selectively affect how negative and positive feedback signals are processed to guide behavior in a reversal learning task. In agreement with our previous findings [41], we found that rats exhibiting high baseline trait anxiety showed greater escalation of cocaine. These rats were also more likely to perseverate with their previous response regardless of whether the outcome was rewarded or not. Importantly, high cocaine escalation rats learned as quickly as control and low cocaine escalation rats from the outcome of each trial but were unable to exploit this information flexibility to adjust behavior when the stimulus-reward contingencies were reversed. These findings support and extend previous findings that cocaine impairs insight and makes actions less sensitive to response outcomes [1, 47] by showing that high rates of cocaine self-administration, associated with trait anxiety, cause a selective disruption in the way negative feedback is used to guide behavior to a food incentive.

Substantial evidence suggests that anxiety can be both a precursor and consequence of drug abuse [48–51], with the perpetuation of drug use possibly reflecting the self-medication of chronic anxiety states [52]. Increased anxiety in rats predicts the propensity to develop a conditioned place preference for cocaine [53], increased oral and intravenous cocaine escalation [40, 41], and increased motivation to self-administer cocaine [39]. These findings were supported by the present study with increased cocaine escalation rates in highly anxious rats particularly during the first hour of each session. However, unlike our previous study [42], where anxiety was assessed using an elevated plus maze rather than an open field, we found no relationship between trait anxiety and behavioral flexibility. This discrepancy may reflect the different measures of anxiety used in each case and that only 15% of the variance in perseverative errors was explained by trait anxiety in our earlier study. Whereas trait anxiety in humans has long been associated with a preferential bias toward negative external cues [54, 55], and impaired set shifting [56, 57], deficits in task-switching reportedly only clearly manifest when attentional control is challenged in highly anxious individuals [58–60]. Thus, the low attentional load of serial spatial reversal learning involving intra-dimensional rather than extra-dimensional shifting [61] may have impeded the expected relationship between trait anxiety and behavioral flexibility to have been reliably detected in the present study.

An important objective of this research was to investigate the nature of the widely reported impairing effects of cocaine on the flexibility of goal-directed behavior [17, 28, 62–65]. Our finding that lose-shift behavior is decreased in HE rats who had some reversal experience is consistent with findings in rats exposed to methamphetamine [34, 35] and in human addicts [66–68]. Rats self-administering methamphetamine have also been reported to show impaired learning from unrewarded outcomes, resulting in reduced model-free learning after stimulant treatment [34] and after non-contingent methamphetamine administration [35]. In addition, model-based impairments have been reported in rats during reversal [34] and habitual behavior on reinforced learning tasks has been reported in humans [67]. In the present study we used a model-free learning algorithm to explain performance on a spatial serial reversal task. By assessing reversal learning before and after response-contingent cocaine administration and using the reinforcement learning framework of Q-learning [45], we were able to define the effects of prior history of escalated cocaine intake on behavioral flexibility measured 8 days after the end of cocaine treatment. Our results demonstrate that rats in the control, low, and high cocaine escalation groups learnt from negative and positive feedback on any given trial and appropriately updated internal representations of choice values, as revealed by no significant change in the alpha modelling parameter, both before and after food or cocaine exposure. Nevertheless, important differences became evident in the way the different groups of rats exploited the value assigned to each choice. This was particularly the case for HE rats, which were more likely to perseverate with their previous choice regardless of the received outcomes. As this deficit was not present prior to drug exposure it was likely the consequence of cocaine itself.

Our analysis of lose-shift and win-stay probabilities revealed that those rats more prone to escalate cocaine self-administration subsequently were less likely to switch behavior on trials that were not rewarded in the reversal learning task. This deficit was clearly the consequence of prior cocaine exposure and did not extend to trials with rewarded outcomes. Maladaptive exploration, as indexed by increased beta and kappa values in HE rats, was also significantly associated with decreased lose-shift probabilities (see supplementary table 2), providing a behavioral validation of the modeling parameters. The relationship between lose-shift probabilities and kappa in cocaine and control rats (supplementary table 3) is mathematically plausible since both measures attempt to capture the association between an agent’s choice on a given trial with their choice on the previous trial. These findings reveal a hitherto unreported deficit in behavioral flexibility caused by cocaine that was restricted to rats with a greater propensity to escalate cocaine intake. Notably, this deficit was present 8 days after the last cocaine session, suggesting it may be caused by relatively long-lasting neural changes, consistent with other studies [28, 62, 69].

Previous research has shown that stimulant addiction in humans is associated with increased perseveration on reversal learning tasks [70, 71]. The present findings go some way to explaining the nature of this deficit whilst building on the earlier finding that cocaine affects the utilization of expected reward value to guide behavior [1], possibly due to impaired executive control over action selection. Specifically, our computational analysis revealed that rewarded and non-rewarded trials were differentially exploited in rats with a history of high-escalation cocaine self-administration. The finding that rats that more readily escalate cocaine self-administration do so because they become insensitive to the anxiogenic properties of cocaine is consistent with this account [72–74]. However, it should be noted that whilst anxiety predicted increased rates of cocaine self-administration, anxiety per se did not predict the failure of HE rats to exploit negative feedback to guide behavior. Our findings suggest therefore that interactive effects between trait anxiety and cocaine exposure were somehow responsible for the inability of HE rats to exploit reward value during the reversal session.

Reversal learning has been widely shown to depend on monoaminergic mechanisms in the OFC and striatum [75–80] with substantial evidence implicating D2 receptors in the striatal indirect pathway [81–85]. However, rather than decreasing *DRD2* expression in the striatum, as predicted from prior positron emission tomography imaging studies in humans and other rats, [86–88], striatal *DRD2* expression increased significantly in the DMS after 8 days of abstinence from cocaine in LE and HE rats. This effect has been reported before after-cocaine exposure and may reflect a delayed compensatory rebound in D2 receptor regulation [89–91]. Since *DRD2* expression in the DMS increased in both HE and LE rats, this was presumably the consequence of prior cocaine exposure rather than a contributing factor to the failure of HE rats to utilize outcome value during reversal. However, without additional studies to measure protein levels it is unclear whether increased *DRD2* expression resulted in increased D2 receptor density. By contrast, HE rats exhibited increased *DRD2* and decreased *HTR2C* expression in the ventral striatum, with a corresponding reduction in *DRD1* expression in the OFC. However, one should be cautious about linking these differentially-expressed genes for OFC-striatal circuit function and specifically whether they contributed to the failure of HE rats to exploit previously learnt outcome value, especially as qualitatively similar but statistically non-significant effects were also observed in LE rats. Nevertheless, highly-impulsive rats that subsequently developed persistent cocaine-taking in the face of aversive outcomes [92] also exhibited reduced *HTR2C* expression in the ventral striatum after long-access cocaine SA [93]. Since the 5-HT2C receptor has been shown to modulate learning from negative feedback in the context of reversal learning [94, 95], impaired 5-HT2C receptor transmission may have contributed to the failure of HE rats to process negative feedback in the present study.

### Synthesis and conclusions

The present findings add to the growing body of evidence that cocaine impairs how negative feedback is used to guide behavior. Using traditional and computational methods of analysis, we report that rats exposed to response-contingent cocaine, and which more rapidly escalate intake, were able to learn the value of changing reward contingencies but were compromised in exploiting this knowledge to guide appropriate actions on a serial reversal task. Previous research has shown that the encoding of expected outcomes to acquired values depends on interactions between the OFC and basolateral amygdala [96] and that cocaine disrupts insight into the consequences of behavior by OFC-dependent mechanisms [47]. Abnormalities within this circuitry may thus be relevant to understanding why individuals addicted to drugs persist with drug consumption despite adverse consequences of continued drug use.

## Funding and disclosure

This work was funded by a Medical Research Council (MRC) Programme Grant (G1002231) and by a core award from the MRC (G1000183) and Wellcome Trust (093875/Z/10/Z) to the Behavioural and Clinical Neuroscience Institute at Cambridge University. P.Z. was supported by the Pinsent Darwin studentship from the Physiology, Development, and Neuroscience Department at Cambridge University. J.A. was supported by a Fellowship from the Swedish Research Council. BJ was supported by Fellowships from the AXA Research Fund, the National Health and MRC of Australia, and the Cambridge Newton Trust. J.S. was supported by a studentship from Boehringer Ingelheim Pharma GmbH, Biberach, Germany and a studentship from La Caixa Foundation, Barcelona, Spain. T.W.R. is a consultant for and receives royalties from Cambridge Cognition; is a consultant for and received a research grant from Eli Lilly; received a research grant from GlaxoSmithKline; is a consultant for and received a research grant from Lundbeck; and is a consultant for Teva, Shire Pharmaceuticals, Mundipharma and Otsuka. J.W.D. has received research grants from Boehringer Ingelheim Pharma GmbH and GlaxoSmithKline. The remaining authors declare no competing interests.

## Supplementary information

Supplemental Material

Supplemental Table 1

Supplemental Table 2

Supplemental Table 3
