# Correction: Withdrawal from escalated cocaine self-administration impairs reversal learning by disrupting the effects of negative feedback on reward exploitation: a behavioral and computational analysis

**DOI:** 10.1038/s41386-019-0576-4

**Published:** 2019-12-04

**Authors:** Peter Zhukovsky, Mickael Puaud, Bianca Jupp, Júlia Sala-Bayo, Johan Alsiö, Jing Xia, Lydia Searle, Zoe Morris, Aryan Sabir, Chiara Giuliano, Barry J. Everitt, David Belin, Trevor W. Robbins, Jeffrey W. Dalley

**Affiliations:** 1 Department of Psychology, University of Cambridge, Downing Street, Cambridge, CB2 3EB UK; 2 Behavioural and Clinical Neuroscience Institute, University of Cambridge, Cambridge, CB2 3EB UK; 3 Department of Psychiatry, University of Cambridge, Cambridge, CB2 2QQ UK

**Keywords:** Risk factors, Decision

Correction to: *Neuropsychopharmacology* 10.1038/s41386-019-0381-0, published online 6 April 2019.

Shortly after publication, the Authors noticed that two of the equations in the Methods section contained errors. The original Article contained a typographic error in Model-free Q-learning models 1 and 3. The correct equations are as follows:

*Model-free Q-learning: model 1*

$$P\left( {c_t = L{\mathrm{|}}Q_t\left( L \right),Q_t\left( R \right)} \right) = \frac{{\exp \left( {Q_t\left( L \right){\mathrm{/}}\beta } \right)}}{{\exp \left( {Q_t\left( L \right)/\beta } \right) + \exp \left( {Q_t\left( R \right)/\beta } \right)}}$$

*Model-free Q-learning: model 3*

$$	P\left( {c_t = L{\mathrm{|}}Q_t\left( L \right),Q_t\left( R \right),L_{t - 1},R_{t - 1}} \right) \\ 	\quad= \frac{{{\mathrm{exp}}\left( {Q_t\left( L \right)/\beta + \kappa \ast L_{t - 1}} \right)}}{{\exp \left( {Q_t\left( L \right)/\beta + \kappa \ast L_{t - 1}} \right) + {\mathrm{exp}}\left( {Q_t\left( R \right)/\beta + \kappa \ast R_{t - 1}} \right)}}$$

The correct equations were used for the data analyses performed in the original Article. The analyses, results and conclusions of the paper were not affected by the typographic error. This has now been corrected in both the PDF and HTML versions of the Article.

