# Supplementary material for: Withdrawal from escalated cocaine self-administration impairs reversal learning by disrupting the effects of negative feedback on reward exploitation: a behavioral and computational analysis
Source: Neuropsychopharmacology. 2019 Apr 6;44(13):2163–73. doi: 10.1038/s41386-019-0381-0 (PMC6895115; doi:10.1038/s41386-019-0381-0)

**Withdrawal from escalated cocaine self-administration impairs reversal learning by disrupting the effects of negative feedback on reward exploitation: a behavioral and computational analysis**

Peter Zhukovsky, Mickael Puaud, Bianca Jupp, Júlia Sala-Bayo, Johan Alsiö, Jing Xia, Lydia Searle, Zoe Morris, Aryan Sabir, Chiara Giuliano, Barry J. Everitt, David Belin, Trevor W. Robbins, Jeffrey W. Dalley

**Supplementary figure 1**. Lack of a relationship between reversal learning (‘total trials to criterion’) at baseline and either escalation ratio during the first hour (A) or anxiety (B).


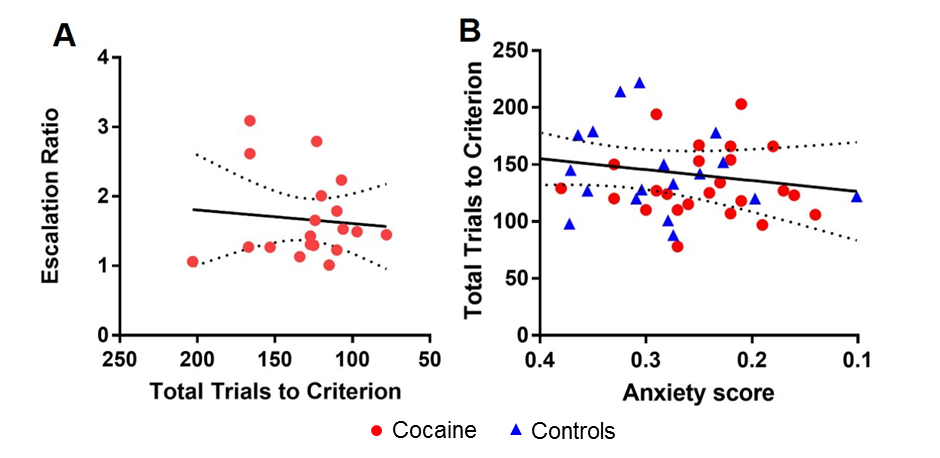

Supplement: Supplementary file 1 — Supplemental Material [file 41386_2019_381_MOESM1_ESM.docx]
