# Supplementary material for: Withdrawal from escalated cocaine self-administration impairs reversal learning by disrupting the effects of negative feedback on reward exploitation: a behavioral and computational analysis
Source: Neuropsychopharmacology. 2019 Apr 6;44(13):2163–73. doi: 10.1038/s41386-019-0381-0 (PMC6895115; doi:10.1038/s41386-019-0381-0)
Supplement: Supplementary file 2 — Supplemental Table 1 [file 41386_2019_381_MOESM2_ESM.docx]

| ***Parameters*** | ***alpha*** | ***alpha No R*** | ***beta*** | ***kappa*** | ***BIC*** | ***pseudo-R^2^*** |
| --- | --- | --- | --- | --- | --- | --- |
| **Pre-Cocaine** |  |  |  |  |  |  |
| Model 1 | 0.15 |  | 0.46 |  | 68.5 | 0.18 |
| Model 2 | 0.33 | 0.07 | 0.33 |  | 69.4 | 0.20 |
| *Model 3* | *0.15* |  | ***0.49*** | *0.28* | *68.8* | *0.21* |
| Model 4 | 0.27 | 0.13 | 0.48 | 0.28 | 71.1 | 0.21 |
| **Post-Cocaine** |  |  |  |  |  |  |
| Model 1 | 0.37 |  | 1.00 |  | 66.3 | 0.13 |
| Model 2 | 0.33 | 0.43 | 0.94 |  | 67.6 | 0.14 |
| *Model 3* | *0.38* |  | ***1.15*** | *0.33* | *66.2* | *0.16* |
| Model 4 | 0.35 | 0.46 | 1.19 | 0.33 | 68.3 | 0.16 |
| **Pre-Food** |  |  |  |  |  |  |
| Model 1 | 0.13 |  | 0.38 |  | 69.3 | 0.21 |
| Model 2 | 0.28 | 0.08 | 0.30 |  | 69.5 | 0.24 |
| *Model 3* | *0.12* |  | *0.37* | *0.16* | *69.9* | *0.23* |
| Model 4 | 0.22 | 0.10 | 0.40 | 0.19 | 71.9 | 0.24 |
| **Post-Food** |  |  |  |  |  |  |
| Model 1 | 0.19 |  | 0.50 |  | 69.2 | 0.18 |
| Model 2 | 0.37 | 0.11 | 0.33 |  | 69.8 | 0.21 |
| *Model 3* | *0.20* |  | *0.52* | *0.22* | *70.3* | *0.20* |
| Model 4 | 0.33 | 0.15 | 0.47 | 0.19 | 72.6 | 0.21 |

Supplementary Table 1: Summary of modelling parameters and model fit using Bayesian Information Criterion (unbiased) and pseudo-R^2^ values (biased towards models with greater number of free parameters). All values are means across the cocaine and the control groups. Model 1 included alpha and beta; Model 2 included alpha-reward and alpha-no-reward; Model 3 included alpha, beta and kappa and Model 4 included alpha-reward, alpha-no-reward, beta and kappa. Lower BIC values indicate better model fit. Model 3 (alpha, beta, kappa) was chosen as it provided better fit than Model 2 to the cocaine group’s data (alpha-reward, alpha no reward) and due to our *a priori* interest in choice autocorrelation (kappa).
