# Supplementary material for: Withdrawal from escalated cocaine self-administration impairs reversal learning by disrupting the effects of negative feedback on reward exploitation: a behavioral and computational analysis
Source: Neuropsychopharmacology. 2019 Apr 6;44(13):2163–73. doi: 10.1038/s41386-019-0381-0 (PMC6895115; doi:10.1038/s41386-019-0381-0)
Supplement: Supplementary file 4 — Supplemental Table 3 [file 41386_2019_381_MOESM4_ESM.docx]

|  | **2.TTC** | **3.PE** | **4.TEC** | **ER** | **5.L-S** | **6.W-S** | **7. α** | **8.β** | **9. κ** | **OFC**  **D1** | **OFC**  **D2** | **OFC**  **2A** | **OFC**  **2C** | **OFC**  **MAOA** | **OFC**  **MAOB** | **VS**  **D1** | **VS**  **D2** | **VS**  **2A** | **VS**  **2C** | **VS**  **MAOA** | **VS**  **MAOB** | **DMS**  **D1** | **DMS**  **D2** | **DMS**  **2A** | **DMS**  **2C** | **DMS**  **MAOA** | **DMS**  **MAOB** |
| --- | --- | --- | --- | --- | --- | --- | --- | --- | --- | --- | --- | --- | --- | --- | --- | --- | --- | --- | --- | --- | --- | --- | --- | --- | --- | --- | --- |
| 1.Open Field | 0.1 | -0.2 | -0.1 |  | -0.2 | -0.1 | 0.2 | 0.2 | 0.2 | 0.2 | 0.4 | -0.1 | 0.0 | 0.2 | 0.0 | ***-0.4*** | -0.2 | -0.2 | -0.2 | 0.2 | 0.2 | -0.1 | -0.4 | 0.4 | -0.2 | -0.2 | -0.3 |
| 2.Total Trials to Crit. |  | **0.7** | **0.9** |  | 0.0 | -0.1 | -0.2 | -0.1 | 0.1 | -0.3 | -0.2 | 0.1 | -0.2 | ***0.6*** | ***-0.5*** | 0.2 | 0.1 | 0.0 | 0.0 | 0.0 | -0.1 | ***-0.5*** | 0.1 | -0.2 | -0.1 | -0.4 | 0.0 |
| 3.Perseverative Errors |  |  | **0.9** |  | 0.1 | -0.1 | -0.2 | -0.2 | -0.1 | -0.4 | -0.2 | 0.0 | -0.1 | 0.2 | -0.4 | 0.1 | 0.1 | -0.3 | -0.2 | -0.1 | -0.3 | -0.4 | 0.0 | -0.2 | -0.2 | ***-0.4*** | 0.0 |
| 4.Total Errors to Crit. |  |  |  |  | 0.2 | -0.3 | -0.2 | -0.2 | -0.3 | -0.3 | -0.2 | 0.0 | -0.3 | 0.4 | ***-0.5*** | 0.1 | 0.1 | -0.1 | -0.2 | 0.0 | -0.2 | -0.3 | 0.1 | -0.2 | -0.2 | -0.4 | 0.0 |
| Escalation ratio |  |  |  |  |  |  |  |  |  |  |  |  |  |  |  |  |  |  |  |  |  |  |  |  |  |  |  |
| 5.Lose-Shift Pr. |  |  |  |  |  | -0.3 | 0.4 | 0.3 | **-0.6** | -0.3 | -0.3 | 0.2 | -0.4 | 0.0 | -0.1 | 0.3 | **0.6** | 0.4 | 0.2 | 0.2 | 0.0 | 0.0 | 0.1 | 0.0 | 0.0 | 0.3 | 0.1 |
| 6.Win-Stay Pr, |  |  |  |  |  |  | 0.2 | 0.3 | ***0.4*** | 0.0 | 0.1 | 0.3 | 0.5 | -0.2 | 0.4 | 0.0 | 0.2 | 0.1 | 0.3 | ***-0.5*** | ***-0.4*** | -0.2 | 0.3 | -0.2 | ***0.5*** | 0.3 | 0.3 |
| 7.Alpha |  |  |  |  |  |  |  | **0.9** | 0.0 | -0.2 | -0.1 | **0.6** | 0.2 | -0.1 | 0.1 | 0.0 | 0.2 | 0.1 | 0.3 | -0.1 | 0.0 | -0.2 | -0.1 | 0.0 | 0.1 | 0.3 | 0.3 |
| 8.Beta |  |  |  |  |  |  |  |  | 0.2 | -0.2 | -0.2 | **0.6** | 0.2 | -0.1 | 0.0 | 0.2 | 0.3 | 0.2 | 0.3 | -0.1 | -0.1 | -0.3 | -0.2 | -0.2 | 0.1 | 0.4 | 0.4 |
| 9.Kappa |  |  |  |  |  |  |  |  |  | 0.2 | 0.1 | -0.2 | 0.2 | -0.1 | -0.2 | 0.1 | 0.0 | 0.0 | 0.4 | -0.2 | 0.1 | -0.4 | -0.1 | -0.2 | -0.1 | -0.1 | -0.1 |

Supplementary Table 3. Correlation table for the control group. Variables included were 1. Open field anxiety scores at baseline assessment; post-cocaine reversal measures including 2. Total trials to criterion3. Perseverative errors, 4. Total errors to criterion; 6. Lose-Shift probabilities, 7. Win-Stay probabilities, 8. Alpha, 9. Beta, and 10. Kappa. Further, D1, D2, HTR2A and HTR2C as well as MAO-A and MAO-B mRNA expression levels in the OFC, VS and DMS were included. Significance is indicated as follows: p<0.01 underlined and bold; p<0.05 italicized and bold. mRNA correlations were Bonferroni corrected; none of the correlations survived multiple comparison corrections, although we do highlight strong trend correlations using uncorrected p-value key. No multiple comparison corrections were applied for behavioral correlations due to presence of strong *a priori* hypotheses.
